# Drastic Reduction of Bacterial, Fungal and Viral Pathogen Titers by Cuprous Oxide Impregnated Medical Textiles

**DOI:** 10.3390/jfb12010009

**Published:** 2021-02-01

**Authors:** Gadi Borkow, Rachel Salvatori, Vikram K. Kanmukhla

**Affiliations:** 1 Cupron Scientific Ltd., Hasadnaot 10, Herzliya 4672837, Israel; gadi@cupron.com; 2 Cupron Inc., 4329 November Avenue, Richmond, VA 23231, USA; rsalvatori@cupron.com

**Keywords:** cuprous oxide, medical textiles, coronavirus, pathogens, hospital acquired infections

## Abstract

Hospital patients and personnel are at risk of nosocomial viral infections, as clearly manifested during the COVID-19 pandemic. Transmission of respiratory viral pathogens can occur through contaminated surfaces, including from medical textiles. Copper has potent biocidal properties, and cuprous oxide impregnated medical textiles (CMT) reduce hospital-acquired bacterial infections. In the current study we confirm the antimicrobial properties of CMT and determine their capacity to reduce infectious titres of human coronavirus (HCoV-229E) in an independent laboratory. The antibacterial and antiviral activities of the CMT were determined according to AATCC TM100-2019 and ISO 18184:2019 standards, respectively. The CMT reduced by 4 logs the viable titers of MRSA, *Klebsiella pneumoniae*, *Enterococcus faecalis*, and *Candida auris* after 2 h of incubation. Viable titers of *Clostridium difficile* were reduced by 2.3, 3, and 4 logs after 2, 6, and 18 h, respectively. Infectious titers of HCoV-229E exposed to CMT for 2 h were reduced by 2.8 and 4 logs (99.85% and 99.99% reductions) as compared to Time-0 control and initial inoculum, respectively. The CMT retain their antibacterial efficacy even after 100 industrial washings. Use of cuprous oxide impregnated textiles in clinical settings may reduce not only hospital acquired infections caused by bacterial and fungal pathogens, but also, and equally important, those caused by coronavirus and other viruses.

## 1. Introduction

Infection from severe acute respiratory syndrome coronavirus 2 (SARS-CoV-2), responsible for the COVID-19 pandemic, can occur through direct exposure to aerosol particles and droplets generated during coughing, sneezing or talking, both by symptomatic and asymptomatic individuals [1,2], or indirectly by touching contaminated surfaces [3]. Unfortunately, SARS-CoV-2 can remain infectious on inanimate surfaces from hours to days [4]. On fabrics the virus can remain infectious for up to 2 days [5]. Similarly, other coronaviruses, as well as other enveloped and non-enveloped viruses, can remain infectious for days on inanimate surfaces [6,7,8].

Copper has potent biocidal properties [9]. Recently the capacity of copper surfaces to reduce the infectious titers of SARS-CoV-2 has been reported [10,11]. No viable SARS-CoV-2 was found on metallic copper after 8 h, while on cardboard, plastic, and stainless steel, viable SARS-CoV-2 could be found even after 24, 72 and 72 h, respectively [11]. Similar higher susceptibility to copper than to all other tested surfaces was also found for SARS-CoV-1 [11].

Significantly, it has been shown that cuprous oxide (Cu_2_O), which primarily forms on copper under ambient conditions, and copper ions released from the metallic copper, are the active forms that cause the inactivation of the viruses and bacteria that come in contact with metallic copper surfaces [12,13,14]. Furthermore, it has been shown that monovalent copper ions (Cu^+^) are much more potent than divalent copper ions (Cu^++^) in their biocidal action in a wide range of tested conditions [15]. In general, the redox cycling between Cu^++^ and Cu^+^ catalyze the production of highly hydroxyl radicals that can damage microbial biomolecules, such as lipids, proteins, and DNA [16]. Under ambient conditions copper oxidizes to cuprous oxide, generating monovalent copper ions [17,18].

Impregnating fibers, yarns, and woven and non-woven textiles with cuprous oxide microparticles endows these products with wide spectrum biocidal properties [19,20]. Woven cuprous oxide impregnated medical textiles (CMT), such as sheets, patient gowns, blankets, and towels, already being used in at least 25 hospitals and in different clinical settings, have been found to reduce hospital acquired bacterial infections [21,22,23,24,25]. In the current study we confirm the capacity of two generations of textiles impregnated with cuprous oxide microparticles, which are currently in use in clinical settings, to reduce bacterial and fungal pathogens and examine their capacity to reduce the infectious titers of human coronavirus (HCoV-229E). HCoV-229E is one of the viruses responsible for the common cold, and its infection is associated with a range of respiratory symptoms, including pneumonia and bronchiolitis. The significance of this study is the demonstration that CMT can also be an additional significant weapon in the arsenal of measures in the battle against hospital-acquired viral infections.

## 2. Materials and Methods

The study (Project ID 0620-CHJ-01-1) was performed according to the ISO Standard 18184:2019-Textiles—Determination of antiviral activity of textile products by Microbial Product Test Laboratory, Situ Biosciences LLC, Wheeling, IL, USA (https://www.situbiosciences.com/).

The following human pathogens were tested: Methicillin resistant *Staphylococcus aureus* (MRSA; ATCC-33592, ATCC (American Type Culture Collection), Manassas, VA, USA); *Staphylococcus aureus* (ATCC-6358); *Klebsiella pneumoniae* (ATCC-1706); *Enterococcus faecalis* (ATCC-51299); *Clostridium difficile* (ATCC-43593); *Candida auris* (ATCC-11903); *Enterobacter aerogenes* (ATCC-13408), and human corona virus (HCoV-229E, ATCC-VR-740).

**Figure 1:**
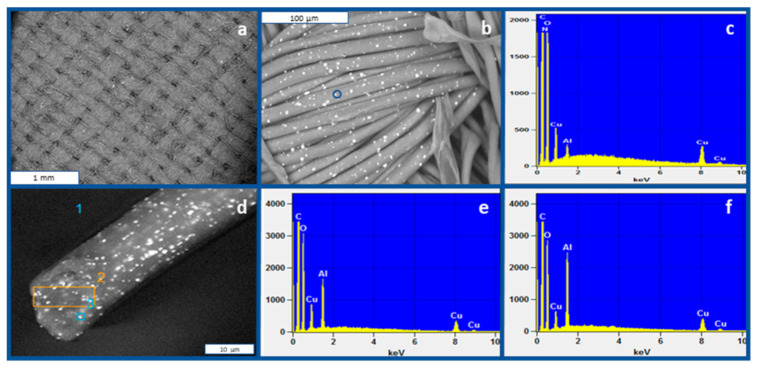
Two scanning electronic microscope (SEM) images of Test Sample 1 (**a**,**b**). (**c**) A representative Energy Dispersive X-ray Analysis (EDX) of a white dot (circled in (**b**)). (**d**) Cross section image of a single fiber showing the cuprous oxide microparticles throughout the fiber. (**e**) EDX analysis of the single-circled white dot shown in (**c**). (**f**) EDX analysis of the square area shown in (**c**). All EDX analyses show a peak at 8 keV corresponding to copper.

Two medical textile fabrics, used to make hospital medical textiles, were tested—CMT Gen 1 and CMT Gen 2, hereafter referred to as Test Sample 1 and Test Sample 2. Test Sample 1 is a 3.2 Oz/Sy plain woven fabric made of 55% cotton and 45% 150D/48 polyester filament impregnated with 2.6% cuprous oxide microparticles weight/weight (*w*/*w*) in the warp (Figure 1). The final concentration of the cuprous oxide in Test Sample 1 is 1.17% w/w. Test Sample 2 is a 3.3 Oz/Sy plain woven fabric made of 60% 60:40 CVC (60% Cotton, 40% polyester blend) in the warp and 40% 150D/48F polyester filament impregnated with 2.6% cuprous oxide microparticles in the weft. The total copper oxide of Test Sample 2 is 2.6 × 40% = 1.04% *w*/*w*.

**Figure 2:**
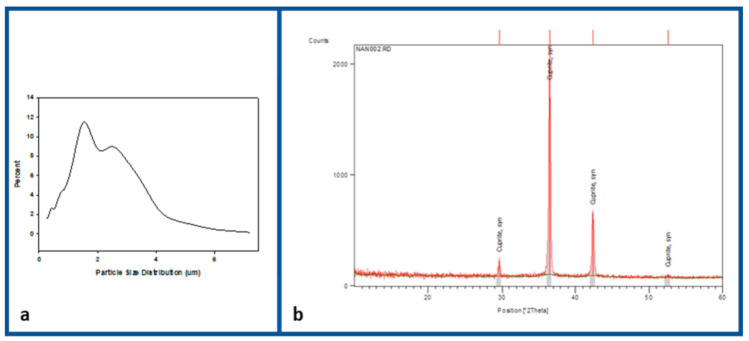
Particle size distribution (**a**) and X-ray diffraction (XRD) analysis (**b**) of the cuprous oxide microparticles.

Figure 2a shows the particle size distribution of the cuprous oxide particles, being the mean size ~2.5 μm. The cuprous oxide particles were further characterized by X-ray diffraction (XRD) using an x-ray diffractometer (Y-2000) with Cu Kα radiation (λ = 1.5418 Å). A scan efficiency of 0.1°·S-1 was applied to record the powder patterns in the range of 0° ≤ 2θ ≤ 60°. Three peaks at 2θ = 29.61°, 36.49° and 42.37° were indexed to (110), (111), and (200) planes of the cubic phase with lattice constant α = 0.4266 nm, which is in accordance with the spectrum for Cu_2_O in JCPDS–International Centre for Diffraction Data (PDF, Powder Diffraction File, No. 05–0667, 1996).

The production of the CMT has been described elsewhere [26]. Briefly, cuprous oxide microparticles in a polyester master batch were added to the slurry of the polyester to a final concentration of 2.6% *w*/*w*. The fibers were then extruded to yield a 150 denier 48 filament yarn. The cuprous oxide microparticles were physically embedded and homogenously distributed throughout the filament yarn. This polyester yarn was then blended with cotton yarns to form the CMT.

As a negative untreated test control material, the same woven fabric but without cuprous oxide was used, hereafter referred as Control Fabric.

The antimicrobial testing was based on the AATCC TM 100-2019. The test bacteria and candida were propagated at 37 °C ± 1 °C in tryptic soy broth overnight. Dilutions of the bacterial suspensions were created with 0.89% NaCl, 5% D/E nutrient broth, and 0.01% Triton X and for *C. auris* in 0.89% NaCl, 5% Sabouraud Dextrose Broth (SDB) and 0.01% Triton X to obtain a final microbial concentration between 1–2 × 10^5^ colony forming units (CFU)/mL.

The Control and Test samples were autoclaved to sterilize them before testing. The samples were then aseptically cut into 1.0 g per piece and laid down onto individual vials. One ml of the microbial test inoculum was pipetted onto the individual fabric samples. Immediately after inoculation, microorganisms from half of the tested samples were recovered by adding 5 mL of D/E neutralizing broth and vortexing for 1 min (Referred to as Time 0). Various dilutions in 0.89% NaCl, 5% D/E nutrient broth, and 0.01% Triton X of the recovered microorganism were plated onto tryptic soy agar in petri dishes. The other half of the tested samples were incubated at 37 °C ± 1 °C and relative humidity of >90% for 2, 6, or 18 h. After the incubation, the microorganisms were recovered from all test samples by adding 5 mL of D/E neutralizing broth and vortexing for 1 min. The recovered microorganisms were plated onto tryptic soy agar in petri dishes. The number of CFU were determined after 48 h of incubation of the plates at 37 °C ± 1 °C and relative humidity of >90%.

The calculations of the microbial titre reductions were determined as follows:

Reduction = % reduction = 100(C − A)/C, where A = the microbial CFU/mL recovered from the inoculated treated test specimen swatches incubated over the contact period (2, 6, or 18 h), and where C = the microbial CFU/mL recovered from the inoculated test specimen swatches immediately after the inoculation.

The antimicrobial efficacy of the fabrics was also tested after 100 washes at a commercial hospital laundry (Handcraft, Richmond, VA, USA). The amount of copper present before and after every 10 washes was determined by inductively coupled plasma optical emission spectrometry (ICP-OES).

The antiviral test was performed according to the ISO 18184:2019 standard. Vero-E6 cells were cultured in DMEM (EM-1) media with antibiotics and 10% fetal bovine serum (FBS) and were inoculated with −80 °C cryopreserved Human Coronavirus 229E (HCoV-229E). The virus infection was monitored by observable cytotoxic effects (CTE). The virus was harvested once the relative percentage of cells affected by CTE was approximately 90%. The virus stock infectivity titer (TCID_50_/mL) was then determined as described below.

The Control and Test samples were autoclaved to sterilize them before testing. The samples were then aseptically cut into 0.4 g per piece and laid down onto individual vials. The samples were then inoculated with 0.2 mL of the viral inoculum at several points on the samples’ surfaces to allow the penetration of the inoculum into the sample fabric. Two time points were created for each test item; Time 0, representing the virus washout of the inoculated sample and Time 2, representing the virus recovered after incubation of the inoculated samples at a temperature of 25 °C (±1 °C) for 2 h. Each control and test sample was prepared in triplicates for each time point. At Time 0 and Time 2 h, 20 mL of the neutralizing solution (soybean–casein digest broth with lecithin and polysorbate 80 medium) was added to the inoculated samples, followed by 1 min of vortexing. Then, aliquots of the neutralizing solutions were used to determine the infective titer of the recovered virus.

In order to control for potential cytotoxicity caused by eluting molecules from the test specimens during vortexing to the neutralizing solution, the following test was conducted: 20 mL of the neutralizing solution was added to non-virus inoculated control and test samples and after vortexing, aliquots from these solutions were tenfold diluted and added to the Vero cells. Cytotoxicity was then monitored. The potential cytotoxicity caused by the neutralizing solution alone at the various dilutions was also examined.

End-point dilutions were conducted with the recovered virus inocula using serial log_10_ dilution factors. TCID_50_/mL (Spearman-Karber; modified by M.A. Ramakrishnan) was then used to determine the concentration of the inoculated virus based on the outcome of the end-point dilution resulting in the CTE of the Vero cells cultured in 96 well plates, as follows:

R = The log 50% end−point dilution = −[(Total CTE/replicate count per dilution) + 0.5] × log dilution factor

where, the total CTE—is the average of the column logarithm of the number of viable virus, in cells/cm^2^, recovered from the untreated test specimens immediately after inoculation (Time 0).

Replicate count per dilution—the number of well replicates inoculated at each dilution.

Log dilution factor—is the dilution factor used for each serial dilution (10x = log_10_(10) = 1).

The antiviral activity value was calculated as follows: log(Va/Vc) = log(Va) − log(Vc), where, log(Va) = the common logarithm average of 3 infectivity titer values immediately after inoculation of the control sample;

log(Vc) = the common logarithm average of 3 infectivity titer values immediately after the 2 h contact time with the tested samples.

Replicate data were utilized in the calculation by the Spearman–Karber method; no additional statistical analysis was conducted.

## 3. Results

Table 1 details the antibacterial and antifungal efficacy of the CMT tested against a panel of human nosocomial pathogens.

A 4-log reduction of the viable titers of the tested pathogens was achieved after 2 h of incubation, with the exception of *C. difficile*, in which a 3 and a 4-log reduction was demonstrated after 18 h of incubation.

As can be seen in Table 2, the antimicrobial efficacy of the Test Samples was maintained even after 100 washes. After 100 washes there was a 1.3% mean loss of copper weight as quantitatively determined by ICP-OES.

Table 3 details the CTE caused by the coronavirus alone (initial inoculum), and after being exposed to the Control and Test Samples for 0 and 2 h, and the CTE caused by the neutralizing solution alone or after being retrieved from the non-virus inoculated control and test samples.

The infectious titer of the viral inoculum was 3.2 × 10^5^ TCID_50_/mL (5.5 log_10_ TCID_50_/mL). The recovered viral titers from the inoculated control and test samples at Time 0 were 2.2 × 10^4^ TCID_50_/mL (4.3 log_10_ TCID_50_/mL). After 2 h of incubation, the infectious titers of the virus were reduced to 3.2 × 10^1^ TCID_50_/mL (1.5 log_10_ TCID_50_/mL) in both Test Samples, i.e., 2.8 and 4 log_10_ reductions (99.853% and 99.99% reductions), as compared to the Time 0 control and initial inoculum, respectively. In contrast, no reduction of the infectious titers occurred in the virus exposed to the Control Sample. No cytotoxicity occurred due to the sample extracted from the fabrics without virus and due to the neutralizer used in all three experiments performed.

## 4. Discussion

Hospital patients and personnel are at risk of nosocomial viral infections [8,27], as clearly manifested during the current COVID-19 pandemic [28,29]. Textiles, especially reusable healthcare textiles that are in close contact with patients for extended periods of time, can significantly contribute to bacterial, fungal and viral nosocomial infections [30,31,32]. It has been found that indirect cross-contamination through transmission of microorganisms, including viruses, which remain infectious on fomite soft and hard surfaces, can contribute to nosocomial infections [33,34].

The capacity of metallic copper and copper alloys to readily neutralize coronaviruses has been demonstrated [14]. Similar infectivity reduction findings were recently reported for SARS-CoV-2 [11]. In the Test Samples, the active copper form is already in the oxidized (activated) form (cuprous oxide). This is one significant step closer to releasing the active copper ions that damage viruses. Importantly, cuprous oxide particles release monovalent copper ions (Cu+), which have been found to be much more potent antivirals and antibacterials than divalent ions (Cu++) [35]. Accordingly, N95 respiratory masks, in which the external layers were made with nonwoven polypropylene fabric impregnated with cuprous oxide microparticles, have been shown to reduce the infectious titers of Human Influenza A (H1N1) and Avian Influenza Virus (H9N2) virions that remain on the mask by more than three logs within 30 min of contact [36]. Similarly, copper oxide impregnated nonwoven fibers and fabrics were shown to reduce infectious titers of 12 additional enveloped and non-enveloped pathogenic viruses [37,38].

The cuprous oxide impregnated woven medical textiles have been in use in medical settings for more than 6 years and 3.4 million patient days, without any adverse effects. The high safety of cuprous oxide impregnated medical and consumer products, such as adult diapers, antimicrobial wound dressings and masks, has been demonstrated in many studies [36,39,40,41]. Cuprous oxide impregnated wound dressings have been cleared by the FDA for the treatment of acute and chronic wounds following extensive biocompatibility studies required for medical devices, demonstrating that they are safe for human tissues.

The use of biocidal textiles can significantly reduce nosocomial infections [30], as demonstrated in several studies in which woven medical textiles, such as sheets, patient gowns, blankets, and towels, impregnated with cuprous oxide microparticles, reduced hospital acquired bacterial infections [21,22,23,24,25]. Indeed, the potent wide spectrum antibacterial and antifungal efficacy of the tested fabrics used to make medical textiles, was demonstrated in the current study. Further, CMT have proven to be durable; even after 100 washes, more than 98% of their copper remained intact, allowing for their continued antibacterial efficacy. Not surprisingly, the cuprous oxide impregnated medical textiles reduced the infectious titers of the HCoV-229E by more than 99% in two hours of exposure, indicating that the use of such textiles in clinical settings may also contribute to the reduction of hospital acquired viral infections.

## 5. Conclusions

The use of cuprous oxide impregnated medical textiles may be an important measure in the arsenal of interventions taken to reduce hospital acquired viral infections. This may be of special significance especially during ongoing pandemics, such as that caused by the current coronavirus SARS-CoV-2, responsible for the COVID-19 pandemic.

## Figures and Tables

**Table 1:** Reduction of viable titers of nosocomial pathogens exposed to the CMT.

Bacteria	Sample	Exposure	Percent	Log
	Time (h)	Reduction	Reduction	
MRSA	Test Sample 1	2	>99.99	>4
	Test Sample 2	2	>99.99	>4
	Control Fabric	2	0	0
*K. pneumoniae*	Test Sample 1	2	>99.98	>3.9
	Test Sample 2	2	>99.99	>4
	Control Fabric	2	0	0
*E. Fecalis*	Test Sample 1	2	>99.99	>4
	Test Sample 2	2	>99.99	>4
	Control Fabric	2	0	0
*C. difficile*	Test Sample 1	2	97.83	1.66
	Test Sample 1	6	>99.91	>3
	Test Sample 1	18	>99.99	>4
	Test Sample 2	2	98.99	2.99
	Test Sample 2	6	>99.91	>3
	Test Sample 2	18	>99.99	>4
	Control Fabric	2/6/18	0	0
*C. auris*	Test Sample 1	2	>99.99	>4
	Test Sample 2	2	>99.99	>4
	Control Fabric	2	0	0

**Table 2:** Antimicrobial efficacy after 100 washes. The initial bacterial titer (CFU) and the titer after 24 h of contact time, and % reduction as compared to the initial inoculum, are detailed.

Test Sample	Contact Time (h)	*Staphylococcus* *aureus*	*Klebsiella* *pneumoniae*	*Enterobacter* *aerogenes*
1	0	6.25 × 10^5^	2.70 × 10^5^	5.65 × 10^5^
	24	3.00 × 10^2^	1.00 × 10^2^	1.00 × 10^2^
	% reduction	>99.9	>99.9	>99.9
2	0	6.25 × 10^5^	2.70 × 10^5^	5.65 × 10^5^
	24	3.00 × 10^3^	1.00 × 10^2^	7.50 × 10^2^
	% reduction	99.52	>99.9	99.87

**Table 3:** Cytotoxicity determinations (CTE) of the various treatments.

	Dilution	Initial	Incubation Time		Controls	
Sample	Factor	Inoculum	0 h	2 h	Extraction	Neutralization
Control Sample	10^−1^	6	6	6	0	0
	10^−2^	6	6	6	0	0
	10^−3^	6	6	6	0	0
	10^−4^	4	5	2	0	0
	10^−5^	2	0	0	0	0
	10^−6^	0	0	0	0	0
	10^−7^	0	0	0	0	0
Test Sample 1	10^−1^	6	6	6	0	0
	10^−2^	6	6	0	0	0
	10^−3^	6	6	0	0	0
	10^−4^	4	5	0	0	0
	10^−5^	2	0	0	0	0
	10^−6^	0	0	0	0	0
	10^−7^	0	0	0	0	0
Test Sample 2	10^−1^	6	6	6	0	0
	10^−2^	6	6	0	0	0
	10^−3^	6	6	0	0	0
	10^−4^	4	5	0	0	0
	10^−5^	2	0	0	0	0
	10^−6^	0	0	0	0	0
	10^−7^	0	0	0	0	0

## Data Availability

The data and independent study reports used for preparing the manuscript are available from the corresponding author on reasonable request.
